# Comparative metabolism of xenobiotic chemicals by cytochrome P450s in the nematode *Caenorhabditis elegans*

**DOI:** 10.1038/s41598-018-31215-w

**Published:** 2018-09-06

**Authors:** Philippa H. Harlow, Simon J. Perry, Alexander J. Stevens, Anthony J. Flemming

**Affiliations:** Syngenta, Jealott’s Hill International Research Centre, Bracknell, Berkshire, RG42 6EY UK

## Abstract

We investigated the metabolic capabilities of *C. elegans* using compounds whose metabolism has been well characterised in mammalian systems. We find that similar metabolites are produced in *C. elegans* as in mammals but that *C. elegans* is deficient in CYP1-like metabolism, as has been seen in other studies. We show that CYP-34A9, CYP-34A10 and CYP-36A1 are the principal enzymes responsible for the metabolism of tolbutamide in *C. elegans*. These are related to the mammalian enzymes that metabolise this compound but are not the closest homologs suggesting that sequence comparison alone will not predict functional conservation among cytochrome P450s. In mammals, metabolite production from amytryptiline and dextromethorphan is dependent on specific cytochrome P450s. However, in *C. elegans* we did not find evidence of similar specificity: the same metabolites were produced but in small amounts by numerous cytochrome P450s. We conclude that, while some aspects of cytochrome P450 mediated metabolism in *C. elegans* are similar to mammals, there are differences in the production of some metabolites and in the underlying genetics of metabolism.

## Introduction

Cytochrome P450s are enzymes with diverse and sometimes essential functions in organisms^1–4^. They primarily catalyse oxidative reactions involving endogenous compounds such as lipids as well as exogenous and xenobiotic chemicals. They have been much studied in pharmaceutical research where they have been found to be important in drug metabolism^5^. They are also important in the evolution of pesticide resistance, particularly in insects^6^. These enzymes are therefore important determinants of the efficacy of both pharmaceuticals and agrochemicals.

Xenobiotic metabolism canonically proceeds through 3 phases^7^. Phase 1 metabolism results in the modification of the xenobiotic, commonly by hydroxylation catalysed by cytochrome P450 enzymes. This makes it a better substrate for Phase 2 metabolism where it is conjugated to a polar moiety, for example the addition of glutathione by glutathione S transferase enzymes. In phase three, the conjugate is excreted by efflux pumps including ATP binding cassette (ABC) transporters.

Phases 1, 2, and 3 of metabolism are well conserved: enzymes required for each phase are found throughout life^5^. However, we wondered whether specific metabolic reactions and the enzymes catalysing them are also conserved i.e. do different species metabolise the same compounds in the same way and, if so, do they use the same enzymes? Theoretically, this provides insight into the evolution of metabolism and whether particular metabolic functions have evolved more than once. Practically, this would indicate whether metabolism by one species could be used as a model for metabolism in another.

Being critical to the first phase of xenobiotic and hence drug metabolism, cytochrome P450s have been extensively studied in mammalian systems whereby particular compounds have been identified as substrates for individual cytochrome P450s and the resulting metabolites identified^8,9^. We used a selection of such compounds and, with a combination of genetics and analytical chemistry, investigated their metabolism in *C. elegans*. Subsequent experiments focussed on the enzymes required for the metabolism of tolbutamide, amitriptyline and dextromethorphan. The results allow a comparative view of the xenobiotic metabolism capabilities of *C. elegans* and mammals informed by the measurement of metabolism itself.

## Results

### *C. elegans* produces metabolites from 7 compounds that are also produced in mammalian systems

We quantified the production of known metabolites from standard substrates in *C. elegans*. The results are shown in Table 1. The metabolites identified in mammalian systems were also recovered in *C. elegans* for all test compounds. However the production of carboxytolbutamide from tolbutamide and the production of paracetamol from phenacetin were only detected at levels close to the limit of quantification suggesting a metabolic deficiency in *C. elegans* versus mammals and relative to the other compounds tested.

**Table 1:** The concentration of known metabolites of the test compounds produced in *C. elegans*.

Compound	Metabolite identified in mammals^9,16^	Mammalian CYP450 required for metabolite production	Metabolite recovered in supernatant (ng/ml)	Metabolite recovered in pellet (ng/ml)	Evidence that this reaction can occur in *C. elegans*
Phenacetin	Paracetamol	CYP1A2	5	14	yes
Tolbutamide	Hydroxytolbutamide	CYP2C8/9/19	1387	276	yes
	Carboxytolbutamide	CYP2C8/9/19	4.5	1	yes
Diclofenac	Hydroxydiclofenac	CYP2C9	67	667	yes
Amitriptyline	Nortriptyline	CYP2C19	207	not tested	yes
	E-10-hydroxyamitriptyline	CYP2D6	40	not tested	yes
Clomipramine	Norclomipramine	CYP2C19	230	1969	yes
Dextromethorphan	Dextrorphan	CYP2D6	1842	1251	yes
	3-methoxymorphinan	CYP3A4	228	250	yes
Nifedipine	Oxidised nifedipine	CYP3A4	692	10725*	yes

We started with 50 µg/ml parent compound in 50 ml flasks over 48 h. Metabolite concentration was then measured after the 50 ml culture had been allowed to settle and concentration was measured by LCMS both in the worm pellet and in the supernatant. The concentration of metabolites produced is expressed in ng/ml. *As this value falls outside the linear calibration range of the experiment (see Methods), it should be taken only to confirm substantial production of the metabolite rather than a precise measure of quantity.

### Tolbutamide metabolism to hydroxytolbutamide is affected by many cytochrome P450s but especially *cyp-34A9, cyp-34A10* and *cyp-36A1*

**Figure 1:**
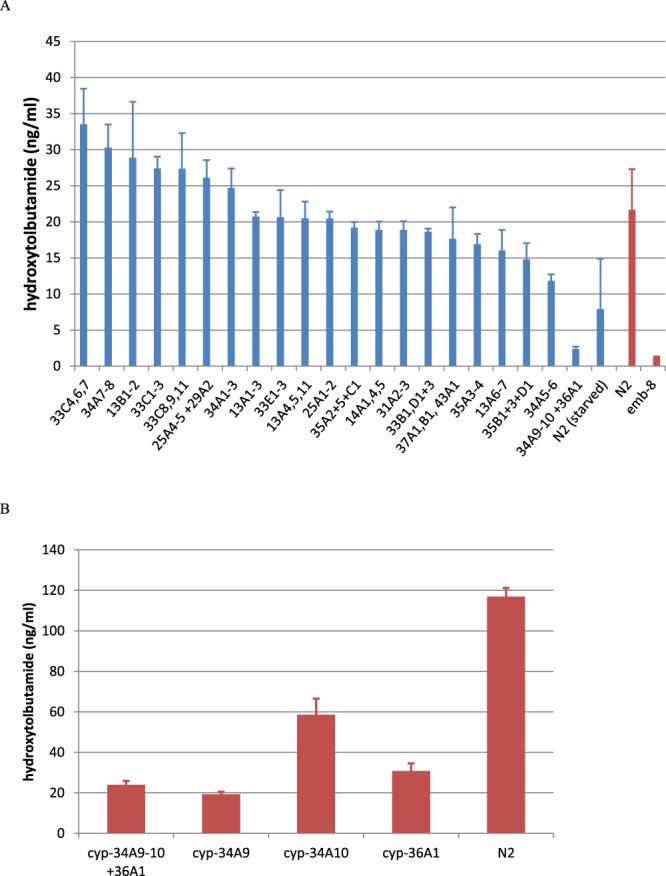
RNAi knockdown of specific cytochrome P450 genes affects the hydroxylation of tolbutamide. Hydroxytolbutamide produced (ng/ml) in the supernatant in response to certain treatment conditions. Error bars show standard error. (n = 3). (**A**) Data in red show N2 (wild type control) compared to *emb-8 (hc69)*, a temperature sensitive strain which was exposed to *emb-8* RNAi and shifted to the restrictive temperature four days before testing. *emb-8* encodes the *C. elegans* cytochrome P450 reductase; under these conditions the mutant strain does not have any detectable NADPH-cytochrome c reductase activity^3^. The *emb-8* animals showed reduced hydroxytolbutamide production (t-test: p = 0.02). Data in blue are a separate experiment showing RNAi knockdown of groups of P450 genes. In this experiment N2 grew faster than anticipated and starved before measurement so metabolism is likely underestimated, indeed N2 versus the *cyp-34A9, cyp-34A10* and *cyp-36A1* group shows no significant difference (Tukey’s multiple comparisons test: p = 0.9998). However, the reduction of metabolism in this RNAi group is substantial compared to other RNAi treatments (e.g. versus the *cyp-25A1-2* group (Tukey’s multiple comparisons test: p = 0.0474) and so, given the starved N2 control, we chose to study this group in a further experiment – see panel B, this figure. (**B**) Simultaneous RNAi knockdown of *cyp-34A9, cyp-34A10* and *cyp-36A1*as a group, RNAi of the same genes individually and N2. In this experiment the assay was modified to ensure no animals starved – see Methods. Compared to N2, all treatments showed significantly reduced metabolite production (Tukey’s multiple comparisons test: p < 0.0001 for all treatments compared to N2). The effects of RNAi of *cyp-34A10* alone was also significantly different to *cyp-34A9* or *cyp-36A1* alone. (Tukey’s multiple comparisons test: *cyp-34A9* vs *cyp-34A10* p = 0.0008, *cyp-34A10* vs *cyp-36A1* p = 0.0095).

Other studies have revealed activity of P450 enzymes by using *C. elegans* strains bearing loss-of-function mutations in the gene *emb-8*^*3*^. The enzyme activity of all P450 genes is dependent on an additional electron transfer step catalysed by cytochrome P450 reductase which is encoded by the *emb-8* gene. Replicating this approach, we find that tolbutamide metabolism to hydroxytolbutamide is a cytochrome P450 dependent reaction by using knockdown of the *emb-8* cytochrome P450 reductase gene. Hydroxytolbutamide was not produced in the *emb-8(hc69)* worms (Fig. 1A). *C. elegans* is thus not capable of hydroxylating tolbutamide without a functional cytochrome P450 reductase. This indicates that this is P450 dependent metabolism but does not specifically indicate which cytochrome P450 enzyme(s) may be involved.

To establish which cytochrome P450 in particular was required for tolbutamide metabolism we used RNAi to knockdown the function of the majority of the *C. elegans* cytochrome P450s. Initially we performed combined RNAi against groups of 2–3 cytochrome P450 genes (See Methods). Where a group showed an effect of interest, the signal was deconvoluted by individual RNAi against constituents of the original group. RNAi was achieved by feeding worms the appropriate strains of *E. coli* for 3 days. Animals were then added to 50 ml liquid cultures containing 50 µg/ml tolbutamide and the concentration of hydroxytolbutamide in the supernatant was measured after 48 h. Unfortunately, an error in experimental design meant that our control treatment starved and the animals were sickly when analysed. Metabolism was likely underestimated because of this (compare with the control treatment for the experiment using *emb-8* described above), the error was corrected for subsequent experiments, see Methods. Despite this, an obvious reduction (>2 fold relative to that seen for any other treatment) in tolbutamide metabolism occurred when the group consisting of *cyp-34A9, cyp-34A10* and *cyp-36A1* was knocked down. Under these conditions less than 3 ng/ml hydroxytolbutamide was produced (Fig. 1A). Many other groups showed a partial reduction in hydroxytolbutamide production, though the significance of these smaller effects is hard to assess versus control in this experiment.

We repeated the experiment for *cyp-34A9, cyp-34A10* and *cyp-36A1* and also targeted each gene separately by RNAi. Hydroxytolbutamide production was reduced to 19% of control when all 3 enzymes were simultaneously targeted. Production was also reduced in response to targeting all three of the genes individually. It was reduced to 15% of control in *cyp-34A9* which was the greatest effect (Fig. 1B). However it was also reduced to 49% of control in *cyp-34A10* and to 25% of control in *cyp-36A1*. This indicates that all three P450s are involved in the metabolism of tolbutamide. As discussed in Methods, we considered that among closely related P450s, RNAi might not be specific. However, the effects of separate RNAi of *cyp-34A9 or cyp-36A1* are both significant relative to *cyp-34A10* (*cyp-34A9* vs *cyp-34A10* p = 0.0008, *cyp-34A10* vs *cyp-36A1* p = 0.0095, Tukey’s multiple comparisons test). This suggests that if non-specific effects are occurring at all, they are not sufficient to obscure effects of individual genes in our experiment. *cyp-34A9, cyp-34A10* and *cyp-36A1* phylogenetically group with mammalian CYP2 of which CYP2C8, 9 & 19 are known to metabolise tolbutamide in mammals.

### Formation of Dextrorphan from Dextromethorphan and Nortriptyline from Amitriptyline in *C. elegans* is affected by knockdown of many cytochrome P450s, but none show marked specificity

**Figure 2:**
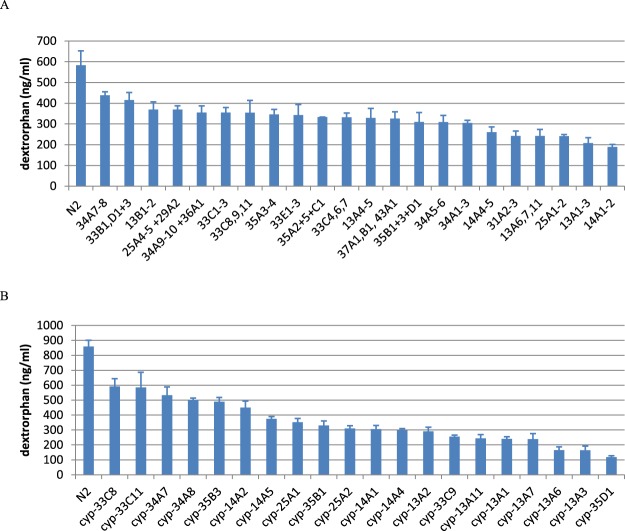
RNAi knockdown of cytochrome P450 genes affects the metabolism of dextromethorphan. Dextrorphan recovered (ng/ml) in the supernatant. Error bars show standard error (n = 3). (**A**) P450s knocked down in small groups. (**B**) P450s knocked down individually.

**Figure 3:**
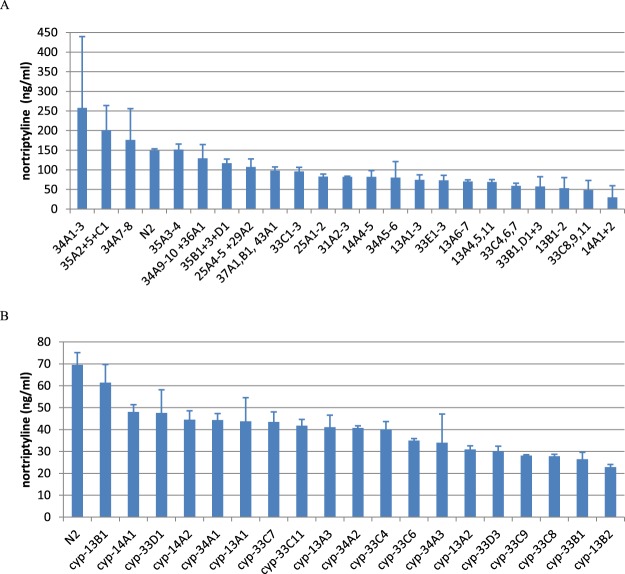
RNAi knockdown of cytochrome P450 genes affects the metabolism of amitriptyline. Nortriptyline produced (ng/ml) in the supernatant. Error bars show standard error (n = 3). (**A**) P450s knocked down in small groups. (**B**) P450s knocked down individually.

Two metabolites were each detected from dextromethorphan (dextrorphan and 3-methoxymorphinan) and amitriptyline (nortriptyline and E-10-hydroxyamitriptyline). We focussed on dextrorphan and nortriptyline respectively in subsequent experiments. When cytochrome P450 expression was systematically knocked down as for tolbutamide above, many of the groups showed reduced metabolite production (Figs 2A and 3A). However, unlike for tolbutamide, no group showed a greater than 2-fold difference to all other treatments.

To investigate this further, we performed RNAi against individual genes from the groups showing the lowest production of metabolites (Figs 2B and 3B). We observed a similar result, that RNAi targeted at many of the P450s caused a partial reduction in metabolite production but in no case did it cause a > 2 fold inhibition of metabolite production relative to all other treatments.

This suggests that no single or small group of cytochrome P450s is responsible for the metabolism of dextromethorphan to dextrorphan in *C. elegans*. Indeed, P450s related to the mammalian CYP2, 3 and 4 families were found to contribute to the metabolism of dextromethorphan in *C. elegans*, while in mammals metabolism of this compound is considered to be dependent on a CYP2 enzyme (CYP2D6)^10^.

## Discussion

We find that the majority of mammalian cytochrome P450 mediated reactions that we tested, also occur in *C. elegans*. Therefore, based on the 7 compounds we studied, the cytochrome P450-based metabolic capabilities of *C. elegans* are broadly similar to those of mammals. In some cases though, very limited metabolite production was observed suggesting a deficiency relative to mammals. For example, we observed limited metabolism of phenacetin to paracetamol in *C. elegans*, as has been previously reported^8^. Phenacetin is metabolised by CYP1-like enzymes in mammals^9^ of which homologs, identifed by bioinformatic sequence comparison, are conspicuously absent in *C. elegans*^11^. Therefore the functional deficiency in CYP1-like metabolism we observed in *C. elegans* for phenacetin, is consistent with a lack of CYP1-like enzymes encoded in the genome. Consistent with this, benzo[a]pyrene, which is also metabolised only by CYP1 isoforms in mammals to form genotoxic metabolites, also does not produce similar metabolites in *C. elegans*^12^.

Similarly we detected little carboxytolbutamide, as metabolised from tolbutamide by CYPs 2C8/9/19 in mammals. Perhaps, then, *C. elegans* is also deficient in metabolism requiring these enzymes in mammals? Apparently not as hydroxytolbutamide, the other metabolite produced in mammals from tolbutamide by CYPS 2C8/9/19, was produced in *C. elegans*. We conclude that if one reaction involving particular cytochrome P450s in mammals is conserved in *C. elegans*, it should not be assumed that all reactions involving those P450s will be.

For tolbutamide we found that all P450s when knocked down had some effect on the production of hydroxytolbutamide but 3 (CYP-34A9, CYP34A10 and CYP-36A1) had a greater effect: a more than 2 fold difference in metabolite production following RNAi compared to any other P450 in the experiment. The corresponding genes show broad sequence homology to the enzymes which metabolise tolbutamide in mammals, CYP2C8, 9 and 19, in that they all belong to the CYP2-like group. However they are not the closest reciprocal homologs of each other. While these enzymes show some similarity to *cyp-34A9, cyp-34A10* and *cyp-36A1* with BLASTP e-values in the range e^−54^ to e^−61^, there are other *C. elegans* P450s that show greater similarity such as *cyp-33C6* (7.7e^−67^), and conversely the closest human homologue to all three enzymes is CYP2A6 with BLASTP e-values in the range e^−66^ to e^−77^. We conclude that full length sequence comparison alone does not predict the metabolising enzyme in either species.

For dextromethorphan and amitryptyline, we found no striking dependence of metabolite production on specific P450s. Rather all cytochrome P450 enzymes partially reduced the metabolism of these compounds. It is hard to know whether this represents a real difference in metabolic capability against these 3 compounds as RNAi experiments suffer from a potential lack of specificity: we may be knocking down the expression of more enzymes than we think in any given treatment. This is superimposed with the possibility of real, functional redundancy among some P450s. That said, we were able to distinguish particular enzymes involved in tolbutamide metabolism, suggesting that our experiment could detect effects of specific P450 knock down. Perhaps our experiment offers a tantalizing glimpse of a more complex metabolic system than one with a simple correspondence between individual P450s and the metabolism of particular compounds. A system where limited metabolism delivered from many P450s combines with, for some compounds but not all, more specific interactions such as those we observed for tolbutamide. Future experiments using CRISPR rather than RNAi would likely resolve this by allowing the production of molecular nulls for each P450, where RNAi delivers knock-down only, and with the possibility of greater specificity^13^.

In conclusion, we show that *C. elegans* has similar, but not identical, metabolic capabilities to mammals for the compounds tested. We concur with other studies that *C. elegans* is deficient in CYP1-like P450 metabolism likely reflecting its lack of CYP1-like P450 enzymes; why CYP1-like enzymes have been lost in the evolution of *C. elegans* is unclear. We add additional cytochrome P450s to the list of those known to be involved in xenobiotic metabolism in *C. elegans* (summarised in Table 2) by showing that tolbutamide is metabolised by *cyp-34A9*, *cyp-34A10* and *cyp36A1*. For dextromethorphan and amitriptyline we find that many cytochrome p450s make only small, incremental contributions to metabolite production. Understanding the metabolic capabilities of target and non-target organisms is important, particularly in non-mammalian animals where metabolism is relatively poorly understood. We demonstrate how analytical chemistry in combination with genetics in *C. elegans* can allow mechanistic insights on measured metabolism in an invertebrate model system and allow comparison with other species. We find differences between *C. elegans* and mammals in metabolic capability and, indeed, even when both species share metabolic capabilities, they are not delivered by the closest cytochrome P450 homologs in each species. This suggests that extrapolation from one species to another should only be done with caution.

**Table 2:** Specific associations between individual cytochrome P450s and metabolite production identified in this and other studies.

*C. elegans* P450 enzyme	Compound	Details	Mammalian Metabolising P450 enzyme	Reference
CYP-35A3 & CYP-35A4	Certain imidazole fungicides	Changes in toxicity		^17^
CYP-34A9 & CYP-36A1 & CYP34A10	Tolbutamide	RNAi knockdown - LCMS	CYP-2C8/9/19	This study
CYP-13A4	Cadmium	Changes in toxicity		^18^
CYP-14A3 & CYP-34A6	PCB52	RNAi knockdown - GCMS	CYP1A1, 2A6, 2A8 and 2B1	^19^
CYP-35A2	Fenitrothion	Changes in toxicity		^20^
CYP-35D1	Thiabendazole	RNAi knockdown – HPLC-UV and changes in toxicity	CYP1A and CYP2B	^21, 22^

## Methods

### Culture of *C. elegans*

The *C. elegans* wild type strain used was Bristol N2. The *C. elegans* strain *emb-8 (hc69)* III MJ69 was used in the *emb-8* experiment. This is a temperature sensitive strain which is wild type at 15 °C and embryonic lethal at 25 °C. The RNAi feeding library used was the *C. elegans* RNAi v1.1 Feeding Library from OpenBiosystems which was derived from the *C. elegans* ORFeome Library v1.1. This is in the form of glycerol stocks of *E. coli* with each strain expressing dsRNA against one *C. elegans* open reading frame. The host *E. coli* strain used for the RNAi treatments was HT115 (DE3). Unless otherwise specified maintenance was according to normal protocols^14^.

### *C. elegans* metabolism assay

Small liquid cultures were prepared containing 50 ml S-medium, 2.5 ml concentrated *E. coli*, 3 large (14 cm) plates of worms and 500 µl compound in DMSO to a final concentration of 50 µg/ml. These bulk cultures were shaken at 20 °C for 2 days. After this they were settled in a 50 ml falcon tube. A supernatant sample was taken and this was added to an equal volume of acetonitrile, vortexed, spun down and the supernatant was transferred to a HPLC vial ready for LC-MS analysis. Samples of the pellet were taken, mixed with an equal amount of acetonitrile and frozen prior to nematode lysis.

### Nematode lysis

Nematode lysis was conducted by the following method: the pellet samples were defrosted at room temperature and the contents of each well were pipetted into an eppendorf, which was frozen in liquid nitrogen and defrosted immediately in the sonicator bath. The samples were homogenised by 2 × 20 s cycles with a FastPrep FP120 (Bio101/Savant) then centrifuged at 10,000 rpm for 15 mins to separate solid debris. The supernatant from each sample was transferred into an HPLC vial and all extracts were analysed by LC-MS. If the extracts could not be analysed immediately they were stored at 4 °C overnight and allowed to warm to room temperature prior to LC-MS analysis. Control samples, containing *C. elegans*, or saline alone were used to make blank controls and calibration curves.

### RNAi knockdown and compound metabolism

Mixed stage populations of *C. elegans* were grown on large (14 cm) agar plates, 3 plates per condition, for three days until the plates were full of worms but not starved. Large agar plates containing 50 µg/ml ampicillin and 1 mM IPTG were seeded with *E. coli* expressing dsRNA from the RNAi library. Initially 5 ml of *E. coli* was used but this was later increased to 20 ml when some worms starved. This *E. coli* was allowed to grow for three days at room temperature and then dsRNA expression was induced by placing the plates overnight at 37 °C. After this the worms were washed in M9 and added to the RNAi plates. Nine RNAi plates were used per condition, three plates for each of three repeats. The worms were left on these plates for three days at 20 °C. They were then washed off and added to 50 ml bulk cultures with compounds and concentrated dsRNA expressing *E. coli* as described above, which were left shaking at 20 °C for 48 h. Expression of dsRNA in the concentrated *E. coli* was induced by IPTG whilst it was grown in shaking flasks at 37 °C. After this the worms were settled and a supernatant sample was taken. This was added to an equal volume of acetonitrile, vortexed and spun down. The supernatant from each sample was transferred into an HPLC vial and analysed by LC-MS. More detailed protocols are provided in supplementary methods. We were concerned that similar P450s, as determined by sequence comparison, might be functionally redundant to one another and so when knocked down separately by RNAi we would fail to observe effects on metabolite production because of compensation from similar, unaffected enzymes. Conversely, we also considered that close sequence similarity might also lead to reduced RNAi specificity and therefore knock down of genes closely related to those targeted; we might therefore fail to distinguish effects of closely related P450s. The extent of knockdown of the genes targeted by RNAi or of other related genes was not quantified directly. Because the RNAi library we used employed the full ORF for each gene, the best design for such an experiment was not obvious and, regardless of this, the large number of measurements that would be required for such a large number of treatments exceeded our available resources at the time. Rather, we performed simultaneous RNAi of groups of P450s belonging to the same subfamily. This increased the likelihood that we would simultaneously knock down redundant enzymes and improve our chance of observing effects on metabolite production. Further, experiments using RNAi against individual enzymes in a group for which an effect on metabolism was observed, would reveal any lack of specificity as we would be unable to resolve differences among the separate RNAi treatments. We designed 21 groups of up to three P450 genes based on homology where possible; feeding worms with mixtures of the corresponding dsRNA producing RNAi bacteria allowed knockdown of multiple genes. Cytochrome P450s are described as being part of the same family (e.g. *cyp-35*) if they share >40% amino acid sequence identity and part of the same sub-family (e.g. *cyp-35A*) if they share >55%^15^. As far as possible the small groups we selected were within the same sub-family. The groups applied together are listed in the Supplementary methods.

### Liquid Chromatography – Mass Spectrometry (LC-MS) Analysis

Reversed-phase high performance liquid chromatography (HPLC) separation was carried out using a ACQUITY UPLC system (Waters, Elstree, UK) and a Waters Atlantis C18 column (5 µm; 150 × 4.6 mm; Waters, Elstree, UK) with a mobile phase mixture of 0.2% formic acid (A) and acetonitrile (B). During the complete 7 minute chromatographic cycle time the linear gradient program was as follows: initial 15% B held for 0.5 min, 15% B increasing to 95% by 4.5 min, 95% B held between 4.5 and 5.5 min, then reduced to 5% B in 0.1 min and 5% B between 5.6 and 7.0 min. The injection volume was 10 µL. A constant flow rate and temperature of 1.0 mL/min and 40 °C, respectively, were maintained throughout the method and the mobile phase was split before reaching the electrospray ionisation mass spectrometry interface.

Mass spectrometry analysis was performed using a Thermo TSQ Vantage instrument (Thermo Fisher, San Jose, US) equipped with an Ion MAX source and HESI-II probe. The operational parameters in positive and negative ionisation modes were as follows: spray voltage (kV) 3.0 (negative 2.2), capillary temperature (°C) 350, auxiliary gas (N2) heater temperature (°C) 270, sheath gas (N2) flow rate (arbitrary units) 55, auxiliary gas (N2) flow rate (arbitrary units) 10 and sweep gas (N2) flow rate (arbitrary units) 0. Mass spectra were obtained by Selected Reaction Monitoring (SRM), the scan parameters were as follows: scan width (s) 0.1, scan time (s) 0.1 and collision gas (argon) pressure (mTorr) 1.5. Stock solutions were used to optimise the SRM transition for each compound and its known metabolite(s), a summary of the LC-MS method is shown in Table 3. Matrix-matched standard solutions of each compound and its known metabolite(s) were analysed alongside the metabolism assay extracts and data processing was performed using Xcalibur (Thermo Fisher, San Jose, US). The matrix-matched standard enabled quantification using the external calibration curves linear concentration range between 2.0 and 2000.0 ng/mL.

**Table 3:** Parameters used in the LC-MS analysis.

Compound	Retention time (minutes)	Ionisation mode	SRM transition	Collision energy
Phenacetin	4.3	Positive	180 > 110	24
Paracetamol	3.1	Positive	152 > 110	24
Tolbutamide	1.8	Negative	269 > 170	20
4-Hydroxytolbutamide	1.1	Negative	285 > 186	20
Carboxytolbutamide	1.2	Negative	299 > 200	19
Diclofenac	nd	nd	nd	nd
4-Hydroxydiclofenac	4.9	Positive	312 > 230	24
Amitriptyline	4.7	Positive	278 > 91	32
Nortriptyline	4.6	Positive	264 > 91	32
Clomipramine	4.2	Positive	315 > 86	24
Norclomipramine	4.1	Positive	301 > 72	24
Dextromethorphan	3.8	Positive	272 > 171	37
Dextrorphan	3.4	Positive	258 > 157	38
3-Methoxymorphinan	3.7	Positive	258 > 215	20
Nifedipine	5.4	Positive	329 > 284*	24
Oxidised nifedipine	5.1	Positive	345 > 284	24

Rows group parent compound (top) and corresponding metabolites. nd = not detected, *parent ion selected results from loss of water from the protonated molecular ion.

## Electronic supplementary material

Supplementary Methods
