# Supplementary material for: Comparative metabolism of xenobiotic chemicals by cytochrome P450s in the nematode Caenorhabditis elegans
Source: Sci Rep. 2018 Sep 6;8:13333. doi: 10.1038/s41598-018-31215-w (PMC6127299; doi:10.1038/s41598-018-31215-w)
Supplement: Supplementary file 1 — Supplementary Methods [file 41598_2018_31215_MOESM1_ESM.docx]

Comparative metabolism of xenobiotic chemicals by cytochrome P450s in the nematode *Caenorhabditis elegans*.

**Philippa H. Harlow, Simon J. Perry, Alex Stevens and Anthony J. Flemming*,**

Syngenta, Jealott’s Hill International Research Centre, Bracknell, Berkshire, RG42 6EY, UK

[*anthony.flemming@syngenta.com](mailto:*anthony.flemming@syngenta.com)

Supplementary Information

Supplementary Information

***C. elegans* metabolism assay protocol**

Day 1 – Pour 14cm plates containing 50ml NGM agar. Nine plates per condition are needed (3 plates per bulk culture and n=3 bulk cultures per condition) plus nine additional plates for the calibration curve.

Make up overnight *E. coli* OP50 cultures in LB.

Prepare sufficient Bristol strain (N2) worms to be able to add well-fed mixed-stage worms to all the 14cm plates on Day 5.

Day 2 – Seed the 14cm plates with 5ml *E. coli* OP50.

Make 5 litres of *E. coli* by adding 1ml of the overnight *E. coli* OP50 cultures to 500ml LB in each of 10x2L conical flasks.

Day 3 – Spin this *E. coli* down and re-suspend in 100ml S-medium to make concentrated *E. coli*. Store in the fridge.

Day 5 – Add N2 worms to the 14cm plates by chunking.

Day 8 – Wash worms off 14cm in S-medium. Prepare small liquid cultures containing 50ml S-medium, 2.5ml concentrated *E. coli*, 3 large (14cm) plates of worms and 500µl compound in DMSO to a final concentration of 50µg/ml. Leave these bulk cultures to shake at 180rpm at 20°C for 2 days. 3 bulk cultures should contain no compound to provide the controls for the calibration curve.

Day 10 – Settle in a 50ml falcon tube in the fridge for 2h. Take a supernatant sample, add this to an equal volume of acetonitrile, vortex, spin down and transfer the supernatant to a HPLC vial ready for LC-MS analysis. Store in fridge.

Take a sample of the pellet, mixed with an equal amount of acetonitrile and freeze prior to nematode lysis.

Day 12 – Nematode lysis

- Defrost the pellet samples at room temperature and pipette the contents of each well into an Eppendorf.
- Freeze this by dropping into liquid nitrogen and defrost immediately in the sonicator bath.
- Homogenise by 2x20s cycles with a FastPrep FP120 (Bio101/Savant), then centrifuge at 10,000rpm for 15mins to separate solid debris.
- Transfer the supernatant from each sample into an HPLC vial ready for analysis by LC-MS. Store at 4°C and allow to warm to room temperature prior to LC-MS analysis.
- Control samples, containing *C. elegans*, or saline alone should be used to make blank controls and calibration curves.

Day 15 – Measure metabolite production by mass spectrometry as described.

**Protocol for measurement of the production of hydroxytolbutamide in worms with a reduction of function in NADPH cytochrome P450 oxidase.**

Day 1 – Pour 14cm plates containing 50ml NGM agar. 27 of these should contain 1mM IPTG and 50µg/ml ampicillin and 9 (starter plates) without. Streak out *emb-8* and control HT115 (DE3) *E. coli* on LB plates containing 100µg/ml ampicillin

Day 2 – Make up overnight cultures of *emb-8* and control RNAi bacteria. Seed starter plates with 5ml OP50 bacteria.

Day 3 – Seed IPTG plates with *emb-8* (9 plates) or control RNAi (18 plates) bacteria

Day 4 – Pick a few worms to each starter plate (*emb-8(hc69)* to 3 plates, N2 to 6 plates), keep at 15°C.

Day 8 – Make RNAi *E. coli* flasks containing IPTG. To do this make up five 2L conical flasks containing 500ml of LB, 1mM IPTG, 50µg/ml ampicillin and 1ml HT115 *E. coli,* per strain. Leave at 37°C overnight.

Day 9 - Make concentrated *E. coli* from these by spinning down, washing and re-suspending the contents of five flasks in 50ml S-media. Keep in the fridge until needed.

Day 11 – Induce IPTG plates at 37°C overnight.

Day 12 – Transfer worms from starter plates to IPTG plates (*emb-8* worms on *emb-8* bacteria and control worms on control bacteria) and move to the restrictive temperature of 25°C.

Day 16 – Wash worms off RNAi plates in S-medium. Add worms to bulk culture with compound at the restrictive temperature. These are 50ml S-media bulk cultures with 2.5ml concentrated *E. coli* and a final concentration of 50µg/ml tolbutamide (added in 500µl DMSO). Three bulk cultures per strain are required as well as three extra controls without compound to make up the controls for the calibration curve. These are then shaken at 180rpm at 20°C for 2 days.

Day 18 – Settle worms in the morning, by pouring into 50ml falcon tubes and leaving in the fridge to settle, and take sample of the supernatant in the afternoon. Vortex samples and add 1 volume acetonitrile to each, vortex, spin down and add 200µl to mass spec vial for LC-MS analysis. Use blank samples to make a calibration curve.

Day 19 - Measure metabolite production by mass spectrometry as described.

**Protocol for RNAi knockdown and compound metabolism for the RNAi library.**

Day 1 – Pour 14cm NGM agar plates containing 50ml NGM agar (starter plates). Three plates are needed per condition to be tested plus three for blank controls.

Day 2 – Seed starter plates with 5ml *E. coli* OP50.

Pour 14cm NGM agar plates containing 50µg/ml ampicillin and 1mM IPTG. Nine plates are needed per condition to be tested plus nine for blank controls.

Day 3 – Streak LB plates containing 100µg/ml ampicillin with *E. coli* from the RNAi library.

Day 4 – Make up overnight cultures of the RNAi bacteria in LB with 100µg/ml ampicillin and leave at 37°C.

Day 5 – Take overnight cultures out and leave in fridge until required. Prepare sufficient Bristol strain (N2) worms to be able to add well-fed mixed-stage worms to all the starter plates on Day 9.

Day 8 – Seed IPTG plates with 20ml RNAi bacteria (HT115 (DE3) *E. coli* expressing dsRNA from the RNAi library) and control and leave at 20°C. Where a treatment is to contain more than one *E. coli* strain to target more than one gene the *E. coli* should be mixed in equal portions prior to seeding. Mixtures used are listed below.

Day 9 – Add N2 worms from a mixed stage population onto the starter plates by chunking and leave at 20°C.

Day 11 – Induce dsRNA expression by placing the RNAi plates overnight at 37°C.

In addition make RNAi *E. coli* flasks containing IPTG. Make up one 2L conical flask containing 500ml of LB, 1mM IPTG, 50µg/ml ampicillin and 1ml of overnight culture of the relevant RNAi bacteria*,* per strain. Leave at 37°C overnight.

Day 12 - The N2 starter plates should be fully populated and just about to run out of *E. coli*. Wash the worms in M9 and add to the RNAi plates. Use nine RNAi plates per condition, three plates for each of three repeats. Leave the worms on these plates for three days at 20°C.

In addition make concentrated *E. coli* from the RNAi flasks by spinning down, washing and re-suspending the contents of each flasks in 10ml S-media. Keep in the fridge until needed.

Day 15 – Wash worms off the RNAi plates in S-medium and add to 50ml S-media bulk cultures with a final concentration of 50µg/ml compound (added in 500µl DMSO) and 2.5ml concentrated dsRNA expressing *E. coli*. Where a treatment is to contain more than one *E. coli* strain to target more than one gene the *E. coli* should be mixed in equal portions prior to adding to the bulk culture.

Leave shaking at 20°C, 180rpm for 48h. Expression of dsRNA in the concentrated *E. coli* is induced by IPTG whilst being grown up in shaking flasks at 37°C. Three plates are used per bulk culture. Three bulk cultures per strain are required as well as three extra controls without compound to make up the controls for the calibration curve.

Day 17 - Settle worms by pouring into 50ml falcon tubes and leaving for 2h in the fridge. Then take a supernatant sample. Add this to an equal volume of acetonitrile, vortex and spin down and add 200µl to mass spec vial for LC-MS analysis. Use blank samples to make a calibration curve.

Day 18 – Measure metabolite production by mass spectrometry as described.

Groupings of P450s used for P450 knockdown

1. *cyp-13A1, cyp-13A2* and *cyp-13A3*
2. *cyp-13A4, cyp-13A5* and *cyp-13A11*
3. *cyp-13A6* and *cyp13A7*
4. *cyp-13B1* and *cyp13B2*
5. *cyp-14A1, cyp-14A4* and *cyp-14A5*
6. *cyp-25A1* and *cyp25A2*
7. *cyp-25A4, cyp-25A5* and *cyp-29A2*
8. *cyp-33C1, cyp-33C2* and *cyp-33C3*
9. *cyp-33C4, cyp-33C6* and *cyp-33C7*
10. *cyp-33C8, cyp-33C9* and *cyp-33C11*
11. *cyp-33D1, cyp-33D3* and *cyp-33B1*
12. *cyp-33E1, cyp-33E2* and *cyp-33E3*
13. *cyp-34A1, cyp-34A2* and *cyp-34A3*
14. *cyp-34A4, cyp-34A5* and *cyp-34A5*
15. *cyp-34A7* and *cyp-34A8*
16. *cyp-34A9, cyp-34A10* and *cyp-36A1*
17. *cyp-35A2, cyp-35A5* and *cyp-35C1*
18. *cyp-35A3* and *cyp-35A4*
19. *cyp-35B1, cyp-35B3* and *cyp-35D1*
20. *cyp-37A1, cyp-37B1* and *cyp-43A1*
